# A telemedicine network for remote paediatric cardiology services in north-east Brazil

**DOI:** 10.2471/BLT.14.148874

**Published:** 2015-09-30

**Authors:** Sandra da Silva Mattos, Sheila Maria Vieira Hazin, Cláudio Teixeira Regis, Juliana Sousa Soares de Araújo, Fernanda Cruz de Lira Albuquerque, Lúcia Roberta Didier Nunes Moser, Thamine de Paula Hatem, Carolina Paim Gomes de Freitas, Felipe Alves Mourato, Thiago Ribeiro Tavares, Renata Grigório Silva Gomes, Rossana Severi, Cícera Rocha Santos, Jailson Ferreira da Silva, Juliana Landim Rezende, Paulo Coelho Vieira, José Luiz de Lima Filho

**Affiliations:** a Círculo do Coração de Pernambuco, Av. Portugal, 163 Paissandu, Recife PE, CEP 52010-010, Brazil.; b Laboratório de Imunopatologia Keiso Asami, Universidade Federal de Pernambuco, Recife, Brazil.

## Abstract

**Problem:**

Providing health care for children with congenital heart diseases remains a major challenge in low- and middle-income countries.

**Approach:**

In October 2011, the Government of Paraíba, Brazil, established a paediatric cardiology network in partnership with the nongovernmental organization Círculo do Coração. A cardiology team supervised all network activities, using the Internet to keep in contact with remote health facilities. The network developed protocols for screening heart defects. Echocardiograms were performed by physicians under direct online supervision of a cardiologist; alternatively, a video recording of the examination was subsequently reviewed by a cardiologist. Cardiovascular surgeons came to a paediatric hospital in the state capital once a week to perform heart surgeries.

**Local setting:**

Until 2011, the State of Paraíba had no structured programme to care for children with heart disease. This often resulted in missed or late diagnosis, with adverse health consequences for the children.

**Relevant changes:**

From 2012 to 2014, 73 751 babies were screened for heart defects and 857 abnormalities were identified. Detection of congenital heart diseases increased from 4.09 to 11.62 per 1000 live births (*P* < 0.001). Over 6000 consultations and echocardiograms were supervised via the Internet. Time to diagnosis, transfers and hospital stays were greatly reduced. A total of 330 operations were carried out with 6.7% (22/330) mortality.

**Lessons learnt:**

Access to an echocardiography machine with remote supervision by a cardiologist improves the detection of congenital heart disease by neonatologists; virtual outpatient clinics facilitate clinical management; the use of Internet technology with simple screening techniques allows resources to be allocated more efficiently.

## Résumé

**Problème:**

Offrir une couverture médicale aux enfants atteints de cardiopathies congénitales reste un problème majeur dans les pays à revenu faible et intermédiaire.

**Approche:**

En octobre 2011, le gouvernement de l'État de Paraíba, au Brésil, a mis en place un réseau de cardiologie pédiatrique en partenariat avec l'organisation non gouvernementale Círculo do Coração. Une équipe de cardiologie a supervisé toutes les activités du réseau, en restant en contact avec les établissements de santé distants grâce à Internet. Le réseau a mis au point des protocoles pour détecter les malformations cardiaques. Des médecins ont réalisé des échocardiogrammes sous la supervision directe en ligne d'un cardiologue; dans d'autres cas, un cardiologue a visionné l'enregistrement vidéo des examens. Des chirurgiens cardiovasculaires se sont rendus une fois par semaine dans un hôpital pédiatrique de la capitale de l'État afin de pratiquer des interventions de chirurgie cardiaque.

**Environnement local:**

Jusqu'en 2011, l'État de Paraíba n'avait pas de programme structuré de prise en charge des enfants atteints de cardiopathies. Cela entraînait souvent des diagnostics erronés ou tardifs, qui avaient des conséquences négatives sur la santé des enfants.

**Changements significatifs:**

De 2012 à 2014, 73 751 bébés ont fait l'objet d'un examen visant à détecter des malformations cardiaques; 957 ont été découvertes. La détection de cardiopathies congénitales est passée de 4,09 à 11,62 pour 1000 naissances vivantes (P < 0,001). Plus de 6000 consultations et échocardiogrammes ont été supervisés via Internet. Les délais de diagnostic, les transferts et les séjours à l'hôpital ont été fortement réduits. Un total de 330 opérations a été pratiqué, avec un taux de mortalité de 6,7% (22/330).

**Leçons tirées:**

L'accès à un échocardiographe avec supervision à distance par un cardiologue améliore la détection des cardiopathies congénitales par les néonatologistes; les cliniques ambulatoires virtuelles facilitent la prise en charge clinique; l'utilisation d'Internet associée à des techniques de détection simples permet une affectation plus efficace des ressources.

## Resumen

**Problema:**

Proporcionar atención sanitaria a los niños con cardiopatías congénitas sigue siendo uno de los principales desafíos en los países de ingresos bajos y medios.

**Enfoque:**

En octubre de 2011, el Gobierno de Paraíba, Brasil, estableció una red de cardiología pediátrica en asociación con la organización no gubernamental Círculo do Coração. Un equipo de cardiología supervisó todas las actividades de la red mediante el uso de Internet para mantenerse en contacto con los centros sanitarios remotos. La red desarrolló protocolos para revisar defectos cardíacos. Los médicos llevaron a cabo ecocardiogramas bajo la directa supervisión online por parte de un cardiólogo; como alternativa, un cardiólogo podía revisar la grabación del examen posteriormente. Los cirujanos cardiovasculares acudían al hospital pediátrico de la capital una vez por semana para llevar a cabo cirugías cardíacas.

**Marco regional:**

Hasta 2011, el Estado de Paraíba no contaba con un programa estructurado para tratar a los niños con enfermedades cardíacas. Esto se traducía en diagnósticos erróneos o tardíos, con consecuencias adversas en la salud de los niños.

**Cambios importantes:**

De 2012 a 2014, se examinaron 73.751 bebés en busca de defectos cardiacos y se identificaron 957 anomalías. La detección de enfermedades cardíacas congénitas subió de 4,09 a 11,62 por cada 1.000 nacimientos (*P*<0,001). Se supervisaron más de 6.000 consultas y ecocardiogramas a través de Internet. Se redujeron considerablemente los tiempos de diagnóstico, traslado y estancias hospitalarias. Se llevaron a cabo 330 operaciones con un 6,7% (22/330) de mortalidad.

**Lecciones aprendidas:**

El acceso a una máquina de ecocardiogramas con supervisión remota por parte de un cardiólogo mejora la detección de enfermedades cardíacas congénitas por parte de neonatólogos; las clínicas ambulatorias virtuales facilitan la gestión clínica; el uso de Internet con simples técnicas de revisión permite que los recursos se distribuyan con mayor eficacia.

## ملخص

**المشكلة:**

يظل تقديم الرعاية الصحية للأطفال الذين يعانون من أمراض القلب الخلقية يمثل تحديًا رئيسيًا في البلدان منخفضة ومتوسطة *  *الدخل.

**الأسلوب:**

أنشأت حكومة بارايبا، في البرازيل شبكة لعلاج أمراض القلب لدى الأطفال بالشراكة مع المنظمة غير الحكومية Círculo do Coração في أكتوبر/تشرين الأول *  *2011. وأشرف فريق أمراض القلب على جميع الأنشطة التي تجريها هذه الشبكة، مع الاستعانة بالإنترنت لاستمرار الاتصال بالمنشآت الصحية النائية. وقد وضعت الشبكة بروتوكولات لفحص عيوب القلب. وتم إجراء عمليات رسم القلب بالموجات فوق الصوتية على يد بعض الأطباء تحت الإشراف المباشر لمختص بأمراض القلب عبر الإنترنت؛ وبديلًا عن ذلك تم اللجوء لمراجعة الفحص بعد تسجيله بالفيديو في وقت لاحق على يد طبيب مختص بأمراض القلب. وقد حضر بعض من جراحي القلب والأوعية الدموية إلى إحدى مستشفيات الأطفال بالولاية العاصمة مرة كل أسبوع لإجراء جراحات *  *القلب.

**المواقع المحلية:**

لم يتوفر في ولاية بارايبا حتى عام *  *2011 أي هيكل منظم لبرنامج يعمل على تقديم الرعاية الصحية للأطفال الذين يعانون من أمراض القلب. وقد نتج عن ذلك في أغلب الأحيان افتقار الحالات إلى التشخيص أو تأخره، إلى جانب حدوث آثار سلبية على الصحة لدى *  *الأطفال.

**التغيرات ذات الصلة:**

تم فحص 73751 *  *رضيعًا للتأكد من وجود عيوب بالقلب وتم تحديد 957 *  *حالة من الاختلال، وذلك في الفترة بين عامي 2012 و2014. وزادت نسبة اكتشاف الحالات المصابة بأمراض القلب الخلقية من 4.09 *  *إلى *  *11.62 بين كل *  *1000 مولود على قيد الحياة (الاحتمال *  *< *  *0.001). وتم الإشراف على ما يزيد عن 6000 *  *استشارة طبية ورسم للقلب بالموجات فوق الصوتية عبر الإنترنت. وقلت إلى حد كبير المدة التي يستغرقها التشخيص ونقل المرضى للمستشفيات وإقامتهم بها. وتم إجراء عدد من الجراحات بإجمالي يبلغ 330 جراحة، بلغت نسبة الوفيات *  *بها *  *6.7% *  *(22/330).

**الدروس المستفادة:**

إن الاستفادة من جهاز رسم القلب بالموجات فوق الصوتية تحت إشراف مختص في أمراض القلب عن بُعد أدى إلى رفع مستوى اكتشاف أمراض القلب الخلقية على يد مختصي طب الأطفال حديثي الولادة؛ كما أن العيادات الخارجية الافتراضية تُيسِّر من الإدارة السريرية؛ فضلًا عن أن استخدام تقنية الإنترنت فيما يتعلق بأساليب الفحص البسيطة يتيح تخصيص الموارد على نحو أكثر *  *فعالية.

## 摘要

**问题:**

在低收入和中等收入国家，为患有先天性心脏病的儿童提供医疗保健服务仍然是一项重大挑战。

**方法:**

在 2011 年 10 月，巴西帕拉伊巴州政府与非政府组织 Círculo do Coração 合作，创立了儿科心脏病网络。心脏病学团队使用互联网与远程医疗设施保持联系，监督所有的网络活动。该网络制定出筛查心脏缺陷的协议。内科医生直接在心脏病专家的在线监督下完成超声波心动图；或者随后由心脏病专家审查检查的视频录像。心血管外科医生一周来州立儿科医院一次，进行心脏手术。

**当地状况:**

在 2011 年之前，帕拉伊巴州没有具备组织性的项目来关怀患有心脏病的儿童。这经常导致错过或延误诊断，给孩子的健康带来不利的结果。

**相关变化:**

从 2012 年至 2014 年，73751 名婴儿被筛查出具有心脏缺陷，且鉴定出 957 例畸形。先天性心脏病的检测从每 1000 名新生儿中 4.09 增加至至 11.62 (*P* < 0.001)。超过 6000 例咨询和超声波心动图通过互联网监督。诊断时间、转移和住院天数均大大降低。共进行了 330 例手术，死亡率为 6.7% (22/330)。

**经验教训:**

通过使用心脏病专家远程监督的超声波心动描记术机器，改进新生儿学专家对先天性心脏病的检测；虚拟门诊促进了临床管理；利用含简单筛查技术的互联网技术，可实现更高效的资源分配。

## Резюме

**Проблема:**

Обеспечение медико-санитарного обслуживания для детей с врожденными заболеваниями сердца остается одной из серьезных проблем в странах с низким и средним уровнем дохода.

**Подход:**

В октябре 2011 года правительство штата Параиба, Бразилия, создало сеть детской кардиологии в сотрудничестве с неправительственной организацией Círculo do Coração. Группа кардиологов осуществляла надзор над всей деятельностью сети, используя Интернет для связи с отдаленными лечебными учреждениями. Сеть разработала протоколы для выявления нарушения сердечной деятельности. Врачи выполняли эхокардиографию под непосредственным контролем кардиологов в режиме онлайн; в качестве альтернативы велась видеозапись обследования, которую затем просматривал кардиолог. Раз в неделю для проведения операций на сердце в педиатрическую больницу столицы штата приезжали врачи, специализирующиеся на сердечно-сосудистой хирургии.

**Местные условия:**

До 2011 года в штате Параиба отсутствовала структурированная программа лечения детей с заболеваниями сердца. Из-за этого диагноз ребенку часто не ставился или ставился с опозданием, что приводило к неблагоприятным последствиям для его здоровья.

**Осуществленные перемены:**

В период с 2012 по 2014 год было проведено обследование 73 751 ребенка с целью выявления заболеваний сердца, в результате которого было обнаружено 957 отклонений. Количество выявленных врожденных заболеваний сердца выросло с 4,09 до 11,62 на 1 000 живорожденных младенцев (*P* < 0,001). С помощью Интернета было проведено более 6000 консультаций и процедур эхокардиографии под наблюдением специалистов. Время постановки диагноза, количество перемещений пациентов и время их пребывания в больнице значительно сократились. В общей сложности было проведено 330 операций, причем уровень смертности составил 6,7% (22 случая из 330).

**Выводы:**

Возможность использования эхокардиографа при дистанционной консультации кардиолога позволяет неонатологам выявить больше врожденных заболеваний сердца; виртуальные амбулатории содействуют клиническому лечению; использование интернет-технологий в сочетании с простыми методиками скринингового обследования позволяет более эффективно распределять ресурсы.

## Introduction

Caring for children with heart defects remains a challenge worldwide.^1^ In developing countries, diagnoses are often late due to the lack of screening programmes and trained personnel.^2^ The problem is worsened by limited availability of hospital beds and the remoteness of rural communities from main urban centres where paediatric cardiology specialists are available.^3^ Brazil faces all of these challenges, particularly in its poorest areas, the north and north-east parts of the country.^4^

## Local setting

**Fig. 1:**
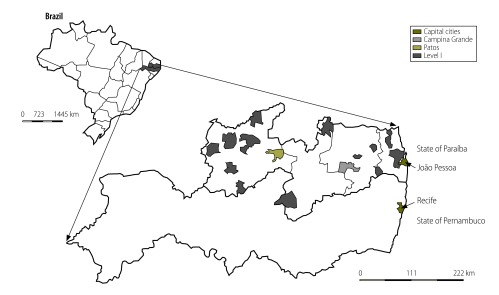
Health facilities in the Círculo do Coração paediatric cardiology network, Paraíba and Pernambuco, Brazil, 2014

Note: Recife and João Pessoa are the capital cities of the States of Pernambuco and Paraíba, respectively. Recife hosts the Círculo do Coração. João Pessoa hosts five network centres: the paediatric hospital, one level II and three level I maternity centres. Campina Grande is the largest city inland and hosts three centres: one level II and two level I. Patos hosts the third level II maternity unit. All other units are level I.

The State of Paraíba, located in north-east Brazil, has 3.7 million inhabitants. Around 70% of the children are cared for by the public health system; many live in rural areas and most come from very poor backgrounds. As there were no established paediatric cardiology facilities in Paraíba, children had to be referred outside the state for diagnosis and treatment. One of the main referral centres is located in the city of Recife, in the neighbouring state, Pernambuco (Fig. 1). Children were referred from towns and villages as far as 500 km from Recife; many arrived after a long time on a waiting list, with consequent deterioration of their clinical condition and some children died before being seen by the specialist.^4^

## Approach

The need to improve this situation was evident and with scant existing resources and personnel, a novel solution had to be devised. Over the last two decades, telemedicine has proved to be an efficient tool for many point-of-care health applications.^5^^,^^6^ In October 2011, a partnership programme was established between the Health Secretary of Paraíba and Círculo do Coração, a nongovernmental organization from Recife.

We conducted a review of routinely collected data^7^ on birth and death rates, socioeconomic conditions and the prevalence of heart defects in children living in Paraíba, from January 2001 to December 2011. Two initial priorities were defined: the establishment of a neonatal screening programme for the whole state and a hospital facility designated to manage patients.

### Network structure

Initially, the 12 largest public maternity centres in the state were selected, together with one paediatric hospital. Centres were divided into three levels (designated I to III). All centres received tablet computers and pulse oximeters (level I); three maternity units also received a portable echocardiography machine (level II) and the paediatric hospital in the capital city of Paraíba State was equipped as a cardiology centre (level III). In 2014, further health centres were included in the network and training and consultation were expanded to include all aspects of perinatology (Fig. 1). A website was developed (https://www.circulodocoracao.com.br/sites/circor/en) and teleconference software was acquired. Three online clinics were established. Their purpose was to allow local paediatricians to examine children with heart defects with guidance from paediatric cardiologists via the Internet. These sessions aimed to reduce travel costs and provide a closer follow-up of children by the network.

A cardiology team was on-call 24 hours per day to supervise all network activities. The team consisted of 7 cardiologists, 3 residents and 4 staff (located in Recife). Three were specialized in paediatric echocardiography. The cardiology team performed daily rounds in all neonatal units from the participating sites, maintained intensive care unit supervision and organized teaching sessions, clinical and surgical meetings. A new perinatology team (with 13 neonatologists) joined the network in 2014. The perinatology team was mostly involved in teaching and seeing patients within the maternity centres. The health professionals were paid for the additional on call time – approximately 2000 United States dollars (US$) per month (exchange rate of 3 Brazilian Real to US$ 1) – by Círculo do Coração with funds from the Government of Paraíba.

### Protocol development

Four initial protocols were developed by Círculo do Coração: (i) a training protocol, to explain the use of all equipment and software; (ii) a focused clinical examination protocol, to remind clinicians about the details of neonatal cardiology examination before discharge; (iii) a protocol for pulse oximetry testing of all babies born after 34-weeks gestation, based on guidelines published at the time^8^; and (iv) a screening echocardiogram protocol for neonatologists, which included three two-dimensional anatomical views and colour flow Doppler imaging.^9^ Members from all units were invited to participate in training sessions to learn and adhere to protocols. Each centre appointed three coordinators (one physician, one nurse and one computer support person) to report results and problems to the reference centre. The training protocol included an initial eight hour course followed by online sessions for all team members.

### Screening tests

Indications for screening echocardiograms were either an abnormal clinical examination or pulse oximetry, defined as an oxygen saturation ≤ 95% or a difference in saturation greater than 2% between the right hand and one foot.^10^ Abnormal pulse oximetry results were automatically noted on a database, allowing the network to contact the clinic and request that they follow up any babies discharged home with abnormal test results. This active search protocol tracked over 80% (59 013/73 751) of the discharged neonates and ensured that abnormal findings were acted on.

Echocardiograms were done by neonatologists under direct online supervision by paediatric cardiologists, or a video recording of the examination was stored and forwarded together with the neonatologist’s initial diagnostic impression. Paediatric cardiologists reviewed and reported on the videos, with advice on clinical management, within one day. Virtual outpatient sessions, ward rounds and other meetings were also scheduled to provide a full range of interactions between the health workers in rural areas and smaller municipalities in Paraíba and the paediatric cardiologists at the reference centre.

Surgeons and anaesthetists from Recife agreed to travel to the paediatric hospital in João Pessoa, the capital city of Paraíba, once a week, to perform heart surgery. The more complex cases, however, were referred to Recife.

### Technical specifications

Internet connections were unreliable for some health centres. To overcome this problem, tablet computers with third generation mobile wireless Internet connections were distributed to all centres. Webex teleconference software (WebEx Communications Inc., Milpitas, California) was acquired to provide secure communication over the Internet. Online meetings were held each day, among all centres, using existing tablets or laptop computers. Echocardiogram images were either directly acquired from the echocardiogram screens or stored and subsequently uploaded to the website.

## Relevant changes

In total, 76 374 patients were seen from January 2012 to December 2014. This included 190 pregnant women (0.2%); 73 751 neonates (96.6%) and 2433 older children (3.2%) with a mean age of 3.04 ± 3.77 years, (range: 30 days to 17.5 years). This represents approximately 80% (73 751/91 615) of the target population (neonates with 34 or more weeks of gestational age in the participant centres) and over 60% (73 751/120 484) of all births in the public health system in the state. There were 1320 abnormal pulse oximetry tests and 1067 abnormal findings on clinical examination of the cardiovascular system; in 77 cases, both pulse oximetry and clinical examination were abnormal.

Initially, all echocardiograms were done with online supervision by the paediatric cardiologist, as part of the neonatologists’ training. After performing about 100 examinations, the quality of images obtained became significantly better and the operators were more confident. At this point, the cardiologists waited for requests for direct online supervision, which dropped progressively until being sought only when pathological findings were suspected. As there are always new neonatologists being trained, this learning process and interaction between teams is a continuous cycle.

There were 1815 screening echocardiogram tests done, of which 848 were abnormal, 957 were normal and 10 were inconclusive. However, 2310 children had indications for a screening echocardiogram. The difference, 495, was mainly due to false-positive oximetry results early in the development of the network. If an echocardiogram was inconclusive, the diagnosis was subsequently established by echocardiography done by a paediatric cardiologist. From the abnormal and inconclusive echocardiograms, 857 demonstrated congenital heart disease (11.62 per 1000 live births). Neither a patent foramen ovale nor an isolated, small, arterial duct was considered a congenital heart defect. However, a clinically significant patent ductus arteriosus was included, coded as transitional circulation. The prevalence of eight major congenital heart defects before and after the introduction of the cardiology network is compared with previously published data in Table 1.^11^^,^^12^

**Table 1:** Birth prevalence of the most common subtypes of congential heart disease for major country groups (1970–2010) and for Paraíba, Brazil (2001–2011 and 2012–2014)

Type of defect	Prevalence of defect per 1000 births								*P*^c^
	Africa	Asia	Europe	North America	Oceania	South America	Paraíba, Brazil		
							2001–2011^a^	2012–2014^b^	
Atrial septal defect	0.35	1.71	1.66	1.71	0.47	0.70	0.17	1.19	< 0.01
Ventricular septal defect	1.40	2.47	2.71	2.42	2.56	1.86	0.71	3.62	< 0.01
Patent ductus arteriosus	0.45	0.67	0.94	0.50	0.45	0.40	1.10	4.53	< 0.01
Pulmonary stenosis	0.28	0.68	0.50	0.41	0.40	0.36	0.27	0.26	0.96
Tetralogy of fallot	NR	0.42	0.33	0.34	0.31	0.37	0.18	0.21	0.83
Coarctation of the aorta	0.06	0.20	0.34	0.30	0.60	0.30	NR	0.17	< 0.01
Transposition of great arteries	0.67	0.18	0.34	0.25	0.38	0.19	0.13	0.21	0.34
Aortic stenosis	NR	0.08	0.25	0.18	0.18	0.08	0.03	0.04	0.90

^a^ Period before implementation of the paediatric cardiology network.

^b^ Period after implementation of the paediatric cardiology network.

^c^  *P*-value of the comparison between Paraíba 2001–2011 and Paraíba 2012–2014.

Data sources: Christianson et al.^11^ and van der Linde et al.^12^

In the five online clinics supervised by the cardiologists in the network, 1092 patients had over 6000 consultations and echocardiograms. A total of 330 operations were done; 285 in João Pessoa and 45 in the referral centre in Recife. There were 30 neonates (9.1%), 65 infants (19.7%), 78 toddlers (23.6%) and 157 older children (47.6%). The overall mortality of 6.7% (22/330) was within the expected range for developing programmes. Mortality risk was related to surgical complexity and clinical condition according to Rach’s score^13^ and a post-operative index.^14^ Time between birth and diagnosis was less than three days in most cases, with a maximum of 647 days (due to the late clinical presentation of milder forms of congenital heart disease). Hospital transfers as well as hospitalization periods were reduced as children did not have to wait to be transferred for echocardiagrams and operations. The virtual clinics were used to facilitate local follow-up for most patients. There were no cases of medical litigation involving the management of children with congenital heart disease.

The total cost for establishing and operating the network was US$ 1.2 million in the first year. With the expansion to a total of 21 centres and perinatology services in 2014, the annual cost increased to US$ 2.0 million. A more detailed study of the economic impact, including the impact of perinatology services, is being conducted. The initial impact of cardiology services was estimated in comparison with the number of patient transfers outside the north-east area, detection rates for congenital heart defects and litigation costs (details are available from the corresponding author).

## Lessons learnt

Several problems were encountered during development of the network, including inadequate equipment, overloaded clinical settings and local changes in nursing staff with insufficient training of new members of staff. The wide range of health workers using the new technologies was another problem. Local training on the equipment was therefore done on a regular basis in addition to the online training. Access to an echocardiography machine by neonatologists with direct online supervision was the most important factor leading to improved diagnosis of congenital heart disease (Box 1). This screening model is similar to others,^15^^,^^16^ but its impact was probably greater, due to the previous lack of paediatric cardiologists in this population.

Box 1 **Summary of main lessons learnt**

- Access to echocardiograph facilities with online supervision improves the detection of congenital heart disease in this rural setting.
- Online outpatient clinics facilitate clinical management.
- The combination of simple screening techniques and diagnostic technology allows resources to be allocated more efficiently.

Clinical care for the children was a big challenge. Online outpatient clinics were a major facilitator of clinical management, by reducing the need for transportation, empowering local physicians and involving other professionals in patient care. However, children requiring surgery had to enter waiting lists to be directed either to the paediatric hospital in the state capital or to Recife. In conclusion, through both live and online collaborative work, local professionals were able to screen, diagnose and treat children with congenital heart disease from remote areas.
